# Evaluation of galectin-3 and titin in cats with a sarcomeric gene mutation associated with echocardiography

**DOI:** 10.14202/vetworld.2024.2407-2416

**Published:** 2024-10-31

**Authors:** Kanokwan Demeekul, Pratch Sukumolanan, Soontaree Petchdee

**Affiliations:** 1 Department of Cardio-Thoracic Technology, Faculty of Allied Health Sciences, Naresuan University, Phitsanulok, Thailand; 2 Department of Veterinary Nursing, Faculty of Veterinary Technology, Kasetsart University, Bangkok, Thailand; 3 Department of Large Animal and Wildlife Clinical Sciences, Faculty of Veterinary Medicine, Kasetsart University, Kamphaeng Saen, Nakorn Pathom, Thailand

**Keywords:** cardiac biomarker, cat, hypertrophic cardiomyopathy, myosin-binding protein C3

## Abstract

**Background and Aim:**

Cardiac biomarkers, such as serum galectin-3 (Gal-3) and titin levels, may be related to cats with sarcomeric gene mutations. This study evaluated cardiac biomarkers and echocardiographic parameters in cats with or without myosin-binding protein C3 (*MYBPC3*) gene mutations.

**Materials and Methods:**

Forty-two healthy cats without cardiac symptoms, including Bengal, Maine Coon, Scottish fold, and Ragdoll cats, were enrolled in this study. Cats were categorized into three groups: Homozygous wild type (n = 17), homozygous *MYBPC3* gene mutation (n = 14), and heterozygous *MYBPC3* gene mutation (n = 11). All recruited cats underwent echocardiography, and blood samples were collected for DNA extraction. DNA sequencing for *MYBPC3* gene mutations at A31P and A74T loci was first examined by Sanger sequencing. The biomarkers of cardiac fibrosis (Gal-3) and myocardial stiffness (titin) were measured by enzyme-linked immunosorbent assay.

**Results:**

Gal-3 levels >250 pg/mL were associated with echocardiographic parameters. However, Gal-3 levels were not significantly different between cats with *MYBPC3* gene mutations and those in the wild-type group. Titin was associated with the left ventricular (LV) thickness and systolic function (r = 0.405, p = 0.013). Qualitative measurement of titin antibodies showed that the highest percentage of these antibodies was found in homozygous wild-type cats. No correlation was found between titin levels and *MYBPC3* gene mutations. Weight was positively associated with interventricular septum (r = 0.312, p = 0.056) and LV wall thickness (LVPW) (r = 0.219, p = 0.187). However, they were not associated with Gal-3 levels.

**Conclusion:**

LVPW was correlated with weight in cats with sarcomeric gene mutations. Serum titin may be an underlying factor for cardiac hypertrophy in cats.

## Introduction

Myosin-binding protein C3 (*MYBPC3*), a cardiac-specific myofilament, is a sarcomeric protein that regulates cardiac contraction in humans and animals [1–5]. Mutations of the *MYBPC3* gene cause familial hypertrophic cardiomyopathy (HCM) [3–9]. Previous studies have documented that single nucleotide polymorphisms (SNPs) of the *MYBPC3* gene, especially A31P and A74T, are potentially associated with HCM in some cat breeds, such as Maine Coon cat [4, 6–9], Ragdoll cat [2], and Bengal cat [10]. According to recent guidelines, HCM is characterized by a diverse phenotype, which can be explained by the genetic variants of this disease [11]. Abnormalities in heart structure and function may be detected by echocardiography and may contribute to the development of cardiomyopathy. Myocardial fibrosis in patients with HCM is significantly correlated with clinical outcomes, disease progression, and heart failure [12].

Galectin-3 (Gal-3) is a novel biomarker of cardiac fibrosis after infarction that is released by the inflammation process [13, 14]. De Couto *et al*. [15] reported that Gal-3 expression is correlated with systolic and diastolic dysfunction in humans. Moreover, the circulating levels of Gal-3 have been accepted as a marker of cardiac fibrosis in a large study involving patients with heart failure [15]. In addition, Gal-3 is associated with a higher risk of developing heart failure [16]. Furthermore, Gal-3 expression is elevated at an early stage of cardiac hypertrophy and is associated with echocardiographic diastolic function parameters [17]. In addition, a canine study model was used to induce heart failure with preserved ejection fraction (HFpEF). Plasma and myocardial Gal-3 levels affect the severity of cardiac diastolic dysfunction [18]. Accumulating data suggest that Gal-3 is a particular biomarker for detecting cardiac hypertrophy and the severity of heart failure in humans and companion animals. Titin, originally called connectin-3, is a massive scaffold protein found in all striated muscles [19]. The titin fragment is a well-known marker of pathological cardiovascular events, such as cardiac hypertrophy and myocardial infarction, in humans [20–22]. In addition, titin provides an elastic and mechanical characteristic to cardiac muscle function [23–25]. During neonatal human heart development, changes in titin expression affect functional transitions and diastolic filling characteristics [23]. Therefore, the implications of titin in sarcomere deficiency include inotropic/chronotropic damage that decreases cardiac function [22]. At present, titin is attracting attention as a biomarker for detecting cardiac function, especially dilated cardiomyopathy (DCM), mainly in humans but less in animals [26–29]. Therefore, titin may be a biomarker of HCM due to function-related hypertrophic response in cats.

Although the effects of Gal-3 and titin on cardiac function-related gene mutations have been extensively documented in human studies, the impact of these two markers on sarcomeric gene mutations in cats has yet to be described. In addition, the levels of Gal-3 and titin in companion cats were not determined.

This study aimed to evaluate cardiovascular circulating biomarkers in cats, both with and without sarcomeric gene mutations, to gain insights into diagnosing HCM at an early stage, before clinical symptoms appear. However, the relationships among echocardiographic parameters, sarcomeric gene mutations, and cardiac biomarkers in cats have not yet been explored. Therefore, this study examined the association between biomarkers and clinical data in cats with and without sarcomeric gene mutations.

## Materials and Methods

### Ethical approval and Informed consent

The Ethics Committee of Kasetsart University (ACKU-62-VET-059) approved the study protocol. All cats were enrolled from the Animal Teaching Hospital Kamphaeng Saen, Faculty of Veterinary Medicine, Kasetsart University, with the owner’s complete informed verbal consent.

### Study period and location

We conducted a retrospective, single-center, randomized controlled trial from September 2022 to December 2023 at Faculty of Veterinary Medicine, Kasetsart University, Kamphaeng Saen Campus

### Animals

Data were collected through convenience sampling. A total of 42 healthy cats without cardiac symptoms, including 19 Bengal, 13 Maine Coon, eight Scottish-fold, and two ragdoll cats, were enrolled in this study. The cats underwent a full physical examination to evaluate their general condition.

### Echocardiography

The cardiovascular system was assessed using echocardiography (General Electric Vivid 5s, Boston, MA, USA), with continuous electrocardiogram monitoring to analyze cardiac function [10]. A 6-MHz probe was used in this study. Echocardiographic images were obtained and stored for offline analysis. Measurements were taken from the right parasternal long- and short-axis views, and the left ventricular (LV) wall structure and function were determined by analyzing images from two-dimensional and M-mode views. LV wall thickness, LV dimensions, and LV function were assessed using M-mode echocardiography to obtain parameters such as interventricular septal thickness in diastole (IVSd) and systole (IVSs), LV end-diastolic diameter (LVIDd), LV end-systolic diameter (LVIDs), LV posterior wall diastolic thickness (LVPWd), and systolic thickness (LVPWs). The left atrium (LA) to aortic root ratio (LA: Aorta [AO]) was calculated using the Swedish method. Diastolic function, indicated by the E/A ratio, was assessed in the left apical four-chamber view using pulsed-wave (PW) Doppler, and pulmonary velocity was measured using the PW Doppler technique.

**Figure-1:**
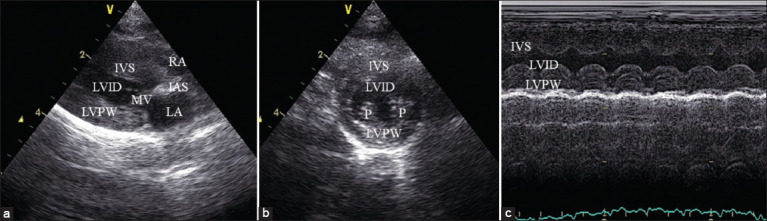
Transthoracic echocardiographic assessment of left ventricular function in the cat. (a) A representative B-mode image obtained from the right parasternal long-axis four-chamber view. (b) Representative B-mode image of the right parasternal short axis at the level of papillary muscle (P) view. (c) Motion mode echocardiography collected from the right parasternal short axis at the level of papillary muscle view. IVS=Interventricular septum thickness, LA=Left atrium, LV=Left ventricular internal dimension, LVPW=Left ventricular posterior wall thickness, P=Papillary muscle.

The LV wall in end-diastole exceeding 0.6 cm indicates LV hypertrophy (LVH) [30]. All echocardiography measurements were performed according to Luis Fuentes *et al*. [11] to minimize the occurrence of variables. Assessments were implemented from stored images; Figure-1a shows B-mode image obtained from the right parasternal long-axis four-chamber view. Figure-1b shows B-mode image of the right parasternal short axis at the level of papillary muscle (P) view. Figure-1c shows motion mode echocardiography from the right parasternal short axis view.

### Polymerase chain reaction (PCR) and DNA sequencing

Fresh blood samples (0.5 mL) were collected from each patient cat for genomic DNA extraction. Blood samples were stored at 4°C for DNA extraction within 24–72 h at the Cardiology Laboratory Unit, Faculty of Veterinary Medicine, Kasetsart University, Kamphaeng Saen Campus. If the blood samples could not be analyzed within 72 h, whole blood in the ethylenediaminetetraacetic acid tube (A-Line, BD, Franklin Lakes, NJ, USA) was kept at −20°C until analysis. In brief, 200 μL of stored blood sample was collected following the manufacturer’s protocol (Favorgen, Taiwan). PCR was conducted for DNA amplification using specific primers for *MYBPC3* gene as described by Godiksen *et al*. [7]. PCR was performed as described by Misaka *et al*. [29]. The nucleotides of the PCR products were determined using Sanger sequencing. The *MYBPC3* gene mutation was detected using the SnapGene Viewer software, version 7.2.1 (https://www.snapgene.com/snapgene-viewer). According to DNA sequencing results, the recruited cats were categorized into three groups: Homozygous wild type (n = 17), homozygous *MYBPC3* gene mutation (n = 14), and heterozygous *MYBPC3* gene mutation (n = 11).

### Measurement of Gal-3 levels in serum

Serum samples stored at −20°C were prepared for the experiments. Quantitative Gal-3 levels in cat serum was measured using a commercially available Gal-3 enzyme-linked immunosorbent assay (ELISA) kit for cats (Cat. No. ab273, Abcam, UK). In summary, 100-fold diluted samples were prepared following the manufacturer’s recommendations. Samples and standard preparation were routinely performed in duplicate. The standard curve was generated by the built-in software and demonstrated linearity. The color intensity was measured at 450 nm using a microplate reader (Infinite F50, Tecan, Switzerland) with Magellan software version 7. X (https://www.tecan.com/knowledge-portal/how-to-register-magellan) [31].

### Detection of titin antibodies in the serum

Qualitative determination of titin antibody was performed in the cats’ serum frozen at −20°C until assayed. The serum level of titin was measured using a commercial Anti-TTN ELISA kit according to the manufacturer’s instructions (Cat. No. MBS104075, MyBioSource, USA). The sample was primarily diluted by serial dilutions from 10 to 100-fold. The suitable dilution was a 100-fold diluted sample. The samples and kit controls were measured in duplicate. The absorbance at 450 nm was measured using a microplate reader (Infinite F50, Tecan, Switzerland) with Magellan software. The detection ranges of titin in the negative and positive controls were typically ≤0.05 and ≥0.15, respectively. The cutoff value was the average optical density (OD) of the negative control wells +0.10 in samples tested by the laboratory.

### Statistical analysis

Data are presented as mean ± standard error of the mean. Normal distribution was calculated using the Shapiro–Wilk test, and sample size was calculated using the Mann–Whitney U test. The comparison of data sets among groups, such as baseline characteristics and echocardiographic variables was performed using a one-way analysis of variance followed by Tukey’s multiple comparison test. A correlation matrix examination was performed to determine the probability metrics of the Gal-3, titin, and echocardiography parameters using GraphPad Prism 9 software (GraphPad Software Inc., San Diego, USA). p < 0.05 indicated a statistically significant difference.

## Results

### Mutations of *MYBPC3* identified by DNA sequencing in recruited cats

**Figure-2:**
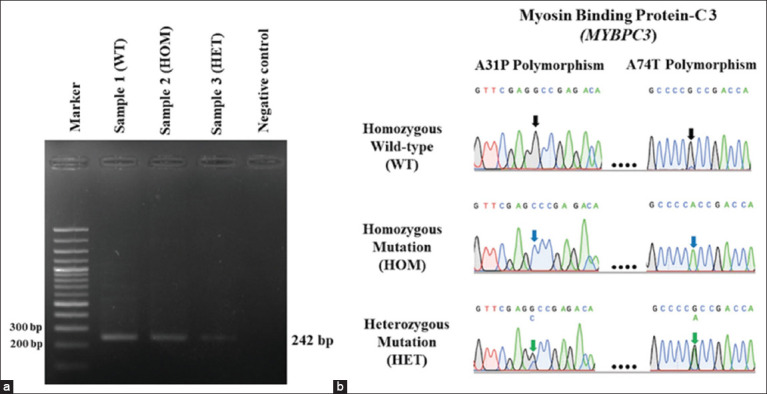
The results of gel electrophoresis and DNA sequencing of the *MYBPC3* gene mutation. (a) Gel electrophoresis revealed 242 bp of target DNA in lanes 2, 3, and 4. (b) *MYBPC3*-A31P and A74T polymorphism DNA sequencing. The dark arrow points to the wild-type group’s guanine (G) peaks. The blue arrow indicates cytosine (C) in the homozygous *MYBPC3*-A31P polymorphism and adenine (A) in the homozygous *MYBPC3-*A74T polymorphism. The green arrow represents two peaks of guanine (G) and cytosine (C) in the homozygous *MYBPC3*-A31P polymorphism and guanine (G) and adenine (A) in the homozygous *MYBPC3*-A74T polymorphism. *MYBPC3=*myosin-binding protein C3.

We performed DNA amplification of *MYBPC3* gene mutations at the A31P and A74T polymorphisms by PCR. The gel electrophoresis analysis indicated that the size of the target band was 242 bp (Figure-2a). Together with the results of DNA sequencing shown in Figure-2b, this analysis showed that homozygous cats for the wild-type genotype (Wild-type group) demonstrated only one peak of guanine (G) for *MYBPC3* gene mutations at both the A31P and A74T polymorphism loci. Furthermore, cytosine (C) and adenine (A) peaks were observed in the cats with homozygous (Homozygous mutation [HOM] group) for the *MYBPC3*-A31P and A74T mutations, respectively. In the heterozygous *MYBPC3*-A31P polymorphism (Heterozygous mutation [HET] group), the peaks of guanine (G) and cytosine (C) are represented. In contrast, guanine (G) and adenine (A) were noticed in the *MYBPC3*-A74T polymorphism. These findings were used to further categorize the animal groups.

### Baseline characteristics of the recruited cats

The recruited cats were divided into three groups (Table-1): Homozygous wild-type group (n = 17), HOM group (n = 14), and HET group (n = 14). The results showed that the cats in the HET group had the highest average age (28.45 ± 6.53 months) except for the lowest weight (4.02 ± 0.23 kg). Most cats in the HOM group were male (71.43%). However, no statistically significant differences in clinical data were observed among the groups.

**Table-1:** Baseline characteristics of all cats.

Parameters (Mean ± SEM)	Wild-type group	HOM group	HET group	p-value
Number/group	17	14	11	-
Age in months	21.12 ± 4.50	18.11 ± 2.90	28.45 ± 6.53	p = 0.07
Weight (kg)	4.17 ± 0.33	4.04 ± 0.37	4.02 ± 0.23	p = 0.94

*p < 0.05 when HOM versus HET; ^#^p < 0.05 when wild-type versus HOM. HET=Heterozygous mutation, HOM=Homozygous mutation, SEM=Standard error of mean

### Echocardiographic variables of the recruited cats

The echocardiography parameters are summarized in Tables-2 and 3. Eight of the 42 cats had ventricular hypertrophy. The eight cats consisted of four Maine Coons and four Bengals, with a median weight of 5.5 kg. One of the Maine Coon cats with LV thickening had a homozygous A31P mutation, and three out of four Bengal cats also had a homozygous A74T mutation. The LA/AO ratio in cats with HOM was significantly elevated compared with the HET (p = 0.02). Moreover, the value of IVSs demonstrated a statistically significant difference among groups (p = 0.01). In comparison, the parameters of IVSd, LVPWd, LVPWs, fractional shortening (FS) percentage, and isovolumic relaxation time (IVRT) were increased in the HOM group compared with the other groups. In contrast, the LVIDd variable was increased in the HET group (p = 0.62). In addition, the LVID variable increased over the reference value in the HET group (p = 0.07). However, no significant differences were observed between the groups.

**Table-2:** Echocardiographic variables of cats grouped by genotype.

Parameters (Mean ± SEM)	Wild-type group	HOM group	HET group	p-value	Reference value
LA/AO ratio	1.32 ± 0.03	1.44 ± 0.05	1.28 ± 0.03*	p = 0.02	0.97–1.39
IVSd (cm)	0.55 ± 0.02	0.57 ± 0.02	0.50 ± 0.04	p = 0.19	0.347–0.621
LVPWd (cm)	0.54 ± 0.02	0.58 ± 0.03	0.56 ± 0.07	p = 0.75	0.343–0.634
IVSd/LVPWd	1.03 ± 0.03	0.99 ± 0.02	0.93 ± 0.05	p = 0.10	0.8–1.4
LVIDd (cm)	1.34 ± 0.09	1.37 ± 0.07	1.46 ± 0.09	p = 0.62	1.076–1.883
IVSs (cm)	0.60 ± 0.02	0.70 ± 0.03^#^	0.58 ± 0.04*	p = 0.01	0.571–1.022
LVPWs (cm)	0.63 ± 0.02	0.68 ± 0.03	0.63 ± 0.05	p = 0.44	0.578–0.989
LVIDs (cm)	1.09 ± 0.07	0.92 ± 0.07	1.28 ± 0.17	p = 0.07	0.495–1.103
FS (%)	37.71 ± 1.14	42.19 ± 1.63	38.74 ± 1.93	p = 0.09	39.9–64.3
IVRT (ms)	49.15 ± 1.95	54.17 ± 2.84	52.50 ± 1.63	p = 0.25	<60

* p < 0.05 when HOM versus HET;

# p < 0.05 when wild-type versus HOM. AO=Aorta, FS=Fractional shortening; HET=Heterozygous mutation, HOM=Homozygous mutation, IVRT=Isovolumic relaxation time, IVSd=Interventricular septum thickness at end-diastole, IVSs=Interventricular septum thickness at end-systole, LA=Left atrium, LVIDd=Left ventricular internal dimension at end-diastole, LVIDs=Left ventricular internal dimension at end-systole, LVPWd=Left ventricular posterior wall thickness at end-diastole, LVPWs=Left ventricular posterior wall thickness at end-systole, SEM=Standard error of mean

**Table-3:** Echocardiographic variables according to the characteristics of cats with left ventricular hypertrophy.

Parameters (Mean ± SEM)	No LVH (n = 34)	LVH (n = 8)	p-value	Reference value
Wild-type group	15	2	-	-
HOM group	10	4	-	-
HET group	9	2	-	-
LA/AO ratio	1.33 ± 0.02	1.39 ± 0.06	p = 0.286	0.97–1.39
LA (cm)	1.04 ± 0.03	1.33 ± 0.06***	p < 0.001	0.96–1.20
IVSd (cm)	0.51 ± 0.02	0.65 ± 0.01****	p < 0.0001	0.347–0.621
LVPWd (cm)	0.52 ± 0.02	0.72 ± 0.06***	p < 0.001	0.343–0.634
LVIDd (cm)	1.39 ± 0.05	1.38 ± 0.13	p = 0.898	1.076–1.883
IVSs (cm)	0.63 ± 0.04	0.75 ± 0.04	p = 0.163	0.571–1.022
LVPWs (cm)	0.61 ± 0.02	0.78 ± 0.04***	p < 0.001	0.578–0.989
LVIDs (cm)	1.46 ± 0.22	0.95 ± 0.04***	p < 0.001	0.495–1.103
FS (%)	40.08 ± 0.96	38.11 ± 2.43	p = 0.419	39.9–64.3
IVRT (ms)	51.04 ± 1.49	55.0 ± 2.28	p = 0.09	< 60

***p < 0.001, ****p < 0.0001 when no LVH versus LVH. AO=Aorta, FS=Fractional shortening, IVSd=Interventricular septum thickness at end-diastole, IVSs=Interventricular septum thickness at end-systole, IVRT=Isovolumic relaxation time, LA=Left atrium, LVH=Left ventricle hypertrophy, LVIDd=Left ventricular internal dimension at end-diastole, LVIDs=Left ventricular internal dimension at end-systole, LVPWd=Left ventricular posterior wall thickness at end-diastole, LVPWs=Left ventricular posterior wall thickness at end-systole, SEM=Standard error of the mean

### Serum Gal-3 level in recruited cats

A reliable ELISA kit (Invitrogen, USA) was used to determine the serum Gal-3. The standard curve correlation coefficient (r) was 0.99997, indicating a strong relationship between concentration and absorbance, indicating that 99.997% of the data can be trusted, whereas 0.003% is an error. The quantitative measurement of serum Gal-3 is presented in Tables-4–6. The average serum Gal-3 level in the population was 204.5 ± 32.59 pg/mL. The highest average serum Gal-3 concentration was observed in cats with the HET mutation (259.23 ± 89.44 pg/mL). Conversely, cats with the HOM mutation had the lowest average concentration of serum Gal-3 compared with the other groups (188.11 ± 29.06 pg/mL). In addition, the average concentration of serum Gal-3 in wild-type cats was 229.39 ± 70.10 pg/mL. Nonetheless, no statistically significant difference was found among all groups (p = 0.77). These findings indicate that Gal-3 levels tend to increase in HET-mutant cats. However, Gal-3 levels were not statistically significant in HET mutations and cats with ventricular hypertrophy (Tables-4 and 5).

**Table-4:** Serum galectin-3 levels in cats grouped by genotype.

Parameters (Mean ± SEM)	Overall population	Wild-type group	HOM group	HET group	p-value
Serum galectin-3 level (pg/mL)	204.5 ± 32.59	229.39 ± 70.10	188.11 ± 29.06	259.23 ± 89.44	p = 0.77

*p < 0.05 when HOM versus HET; ^#^p < 0.05 when wild-type versus HOM. HET=Heterozygous mutation, HOM=Homozygous mutation, SEM=Standard error of the mean

**Table-5:** Serum galectin-3 levels in cats according to the characteristics of cats with left ventricular hypertrophy.

Parameters (Mean ± SEM)	No LVH (n = 34)	LVH (n = 8)	p-value
Serum galectin-3 level (pg/mL)	243.28 ± 40.34	125.51 ± 40.09	p = 0.180

LVH=Left ventricular hypertrophy, SEM=Standard error of the mean

**Table-6:** Serum galectin-3 levels in cats and the echocardiography parameters.

Parameters (Mean ± SEM)	Serum Galectin-3 (0-250 pg/mL) (n = 31)	Serum Galectin-3 (>250 pg/mL) (n = 11)	p-value
LA/AO ratio	1.38 ± 0.03	1.29 ± 0.03	p = 0.10
LA (cm)	1.16 ± 0.04	1.22 ± 0.09	p = 0.54
IVSd (cm)	0.58 ± 0.02	0.55 ± 0.04	p = 0.07
LVPWd (cm)	0.61 ± 0.04	0.61 ± 0.07	p = 0.10
LVIDd (cm)	1.43 ± 0.06	1.35 ± 0.11	p = 0.55
IVSs (cm)	0.68 ± 0.02	0.71 ± 0.10	p = 0.83
LVPWs (cm)	0.69 ± 0.03*	0.63 ± 0.05*	p = 0.02
LVIDs (cm)	1.16 ± 0.10	1.33 ± 0.17	p = 0.14
FS (%)	39.49 ± 1.06*	35.50 ± 0.96*	p = 0.04
IVRT (ms)	53.5 ± 1.12	53.0 ± 2.04	p = 0.88

* p < 0.05 when serum Galectin 3 level 0–250 pg/mL versus > 250 pg/mL. AO=Aorta, FS=Fractional shortening, IVSd=Interventricular septum thickness at end-diastole, IVSs=Interventricular septum thickness at end-systole, IVRT=Isovolumic relaxation time, LA=Left atrium, LVH=Left ventricle hypertrophy, LVIDd=Left ventricular internal dimension at end-diastole, LVIDs=Left ventricular internal dimension at end-systole, LVPWd=Left ventricular posterior wall thickness at end-diastole, LVPWs=Left ventricular posterior wall thickness at end-systole, SEM=Standard error of the mean

### Identification of titin antibodies in recruited cats

The qualitative determination of titin antibodies in serum is presented in Tables-7 and 8. The results showed that 34/42 recruited cats (80.95%) had negative results for detectable titin levels. Furthermore, 8/42 recruited cats (19.05%) were positive for titin detection (OD ≥ 0.15). After categorizing by *MYBPC3* gene mutation, we found that undetectable titin was primarily found in the wild-type group (n = 14, 41.18%), followed by the HOM group (n = 12, 35.29%) and HET group (n = 8, 23.53%). The number of cats in the titin-positive group was equal in the wild-type group and HET as N = 3 (37.50%). Only two cats in the HOM group (25.00%) exhibited positive titin detection for genotype testing. Moreover, 26 cats with negative titin detection had no LVH, while the other 8 cats (100%) with positive titin detection demonstrated LVH (Table-8). Therefore, this study suggested that titin antibody levels were undetectable in the serum of most recruited cats with gene mutations. In contrast, titin antibody levels may indicate LVH.

**Table-7:** Serum titin in cats grouped by genotype.

Detectable-titin level	Overall population (Number [%]) (n = 42)	Wild-type group (Number [%]) (n = 17)	HOM group (Number [%]) (n = 14)	HET group (Number [%]) (n = 11)
Negative (OD < 0.15)	34 (80.95)	14 (41.18)	12 (35.29)	8 (23.53)
Positive (OD ≥ 0.15)	8 (19.05)	3 (37.50)	2 (25.00)	3 (37.50)

HET=Heterozygous mutation, HOM=Homozygous mutation, SEM=Standard error of the mean

**Table-8:** Serum titin in cats according to left ventricle hypertrophy characteristics.

Detectable-titin level	No LVH (Number [%]) (n = 34)	LVH (Number [%]) (n = 8)
Negative (OD <0.15)	26 (76.47)	8 (23.53)
Positive (OD ≥0.15)	-	8 (100)

LVH=Left ventricular hypertrophy

### Correlation matrix of Gal-3, titin, and echocardiography in recruited cats

**Figure-3:**
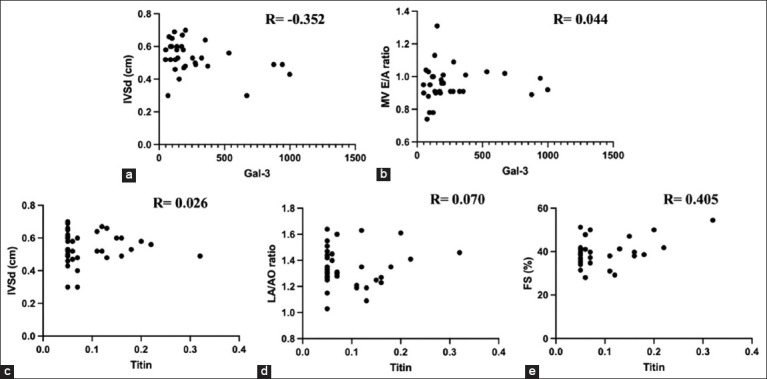
(a) Correlations of galectin-3 and echocardiographic parameter IVSd. (b) galectin-3 and MV E/A ratio. (c) titin and echocardiographic parameter IVSd. (d) titin and LA/AO ratio. (e) titin and percentage of FS among all recruited animals. AO=Aorta, FS=Fractional shortening, Gal-3=Galectin-3, IVSd=Interventricular septum thickness at end-diastole, LA=Left atrium, MV=Mitral valve.

This study generated probability matrices that exhibited correlations between the two cardiac biomarkers and clinical data. Figure-3a shows the correlations of Gal-3 and echocardiographic parameter IVSd. Figure-3b shows correlation between Gal-3 and MV E/A ratio. Figure-3c shows the correlation between titin and echocardiographic parameter IVSd. Figure-3d shows titin and LA/AO ratio. Figure-3e shows titin and percentage of FS. The measurement of serum titin antibodies indicated an imperative point with an increasing probability of echocardiographic parameters, especially the percentage of FS (0.41). In addition, the correlation coefficients of LA/AO, IVRT, and IVSd with titin antibodies were 0.07, 0.07, and 0.03, respectively. However, all echocardiographic parameters showed a weak correlation with serum Gal-3 levels. In addition, the weight of the recruited cats was correlated with increases in the echocardiographic parameters IVSd (0.31) and LVPWd (0.22). In this study, Gal-3 levels >250 pg/mL appeared to predispose the myocardial thickening phenotype; however, only FS and LVPW were statistically significant (p < 0.05) (Table-6).

Table-7 presents the correlation of titin levels with *MYBPC3* gene mutations. This study found no correlation between titin levels and the *MYBPC3* gene mutation. Positive titin levels (OD ≥ 0.15) can be detected in both cats with and without the mutation.

Table-8 shows serum titin levels in cats according to LVH characteristics. All eight cats with LVH had positive titin levels at OD ≥ 0.15. However, all eight cats with LV wall thickening had lower levels of Gal-3 than those with normal LV walls. Gal-3 and titin had weak correlations with the presence of *MYBPC3*-A31P and *MYBPC3*-A74T gene mutations. According to the results of this study, it could be suggested that titin is more strongly associated with cardiac structure than Gal-3, but these two parameters do not appear to be correlated with the presence of *MYBPC3*-A31P and *MYBPC3*-A74T gene mutations. No other variables were statistically significant between the groups, whether or not the absolute values were higher or lower.

## Discussion

This study aimed to verify the circulating biomarkers in cats with or without sarcomeric gene mutations that may provide insights into diagnosing HCM in the early stage before the disease develops into clinical symptoms. This study revealed serum Gal-3 and titin levels in cats with *MYBPC3*-A31P and *MYBPC3*-A74T gene mutations for the 1^st^ time. Although there were no differences in the presence of LVH and the underlying genetic status, the comparison between the two biomarkers demonstrated that serum titin better indicated hypertrophic cardiac disease features in cats than Gal-3.

Previous studies by Yakar Tülüce *et al*. [32] and Stack *et al*. [33] have shown that Gal-3 levels are increased and are correlated with the progression of LVH in patients with HCM, such as the thickness of the interventricular septum and LV mass index. In our study, Gal-3 levels >250 pg/mL appeared to be a predisposition to the phenotypic appearance of myocardium thickening (Table-6). Because of limitations in our study, the Gal-3 assay cannot be used as a good biomarker for detecting HCM in cats. All echocardiography measurements were performed as described by Luis Fuentes *et al*. [11] to minimize the occurrence of variables. Variability can be derived from many factors, such as the variance component for each patient, image acquisition, and reading. The variable from image acquisition is much greater than that from image reading, especially for 2D measurements. Another important variable is the sonographer. However, this study performed echocardiography by a single person certified by the Asian College of Veterinary Internal Medicine (Cardiology).

In addition, a previous study revealed the localization of galectin-3 in phagocytic cups and phagosomes [34]. Therefore, serum Gal-3 levels may not reflect heart tissue deposits in Gal-3. Further studies are required to identify possible mechanisms and levels of Gal-3 that can be used to detect HCM in cats.

Our gene sequencing analysis revealed that more than half of the animals in this study had *MYBPC3* gene mutations in both A31P and A74T (Figure-2 and Table-1). The present study investigated the association between echocardiographic changes and pathogenic gene mutations in cats. The results showed that gene mutations were weakly associated with cardiac echocardiographic parameters (Table-2). Consequently, echocardiographic data showed that more cats had homozygous *MYBPC3* gene mutations than heterozygous *MYBPC3* mutations and wild-type genotypes in the left ventricle hypertrophy group (Table-3). In contrast, the concentration of serum Gal-3 demonstrated a higher trend in cats with heterozygous *MYBPC3* gene mutations, which was sequentially followed by those with wild-type genotypes and homozygous *MYBPC3* gene mutations (Table-4).

In addition, most wild-type cats were negative for titin antibodies (Table-7). However, the correlation analysis suggested that titin was more strongly correlated with echocardiographic parameters than Gal-3 (Figure-3). Therefore, biomarkers, such as titin, can be used to differentiate HCM in cats.

The presence of echocardiographic findings is associated with *MYBPC3* gene mutations. Previous studies [6, 9, 10] support these findings. Sukumolanan and Petchdee reported [9] that SNPs of the *MYBPC3* gene A31P and A74T were responsible for HCM in cats of specific breeds, such as Maine Coon and Bengal. Recently, a study by Nääs [2] focusing on breed-specific gene variants in Ragdoll cats suggested that a variant of the *MYBPC3* gene is a probable cause of the high prevalence of HCM. Of note, our results cannot be directly attributed to the findings of other experiments, as almost all echocardiography variables, except LVIDs and the IVSd/LVPWd ratio, appeared to markedly increase in homozygous cats for *MYBPC3* gene mutations compared with the other groups. Thus, these findings could explain cardiac structure and function changes induced by specific gene mutations.

In our study, the concentration of serum Gal-3 was highest in cats heterozygous for *MYBPC3* gene mutations. This finding can be explained by several underlying mechanisms during the development of cardiovascular events [13, 14]. Due to inflammatory mechanisms considered a major factor in heart failure, Gal-3 is an emerging prognostic biomarker in this disease [33–36]. The difference in Gal-3 levels at the protein expression and circulation levels has been comprehensively revealed in patients with cardiovascular disease. In 2010, Gal-3 was included as a novel marker of cardiac dysfunction [15]. A correlation between Gal-3 expression and systolic and diastolic function impairment was suggested.

Furthermore, a study on human patients reported that circulating Gal-3 levels were affected by heart failure [15]. Moreover, the concentration of Gal-3 was reported in a cohort study [16]. This study found that a higher Gal-3 concentration was correlated with an increased risk of heart failure and mortality incidents in the community. In accordance with this finding, Gal-3 was overexpressed in a rat model of heart failure. Additionally, inflammatory mechanisms may be involved in the development of heart failure in the advanced stage, which relies on Gal-3 expression [17]. Moreover, Wu *et al*. [18] have reported elevated Gal-3 expression before cardiac hypertrophy and that this elevated expression is related to the variable echocardiographic features of diastolic function. In the canine study model, researchers potentially experimented with inducing HFpEF. They mentioned that the plasma and myocardial Gal-3 levels impacted the severity of cardiac diastolic dysfunction. All the accumulating data suggested that serum Gal-3 levels were accepted as a marker of cardiac fibrosis and may provide prognostic information for cardiac hypertrophy and the severity of heart failure.

Based on our results, 20% of all animals were positive for titin antibodies in serum. This qualitative result is consistent with our echocardiography findings. Based on our clinical data, it can be explained that eight of the recruited cats presented with HCM characteristics on echocardiography (Table-3). A comparison of the groups indicated that homozygous *MYBPC3* gene mutations were associated with echocardiographic HCM variables (IVSd, LVPWd, and IVRT). In addition, heterozygous cats for *MYBPC3* gene mutations had high LVIDs and LVIDd values.

As well known, titin is important for sarcomeric structure and function [19, 29]. Focusing on the cardiovascular system, titin was found to be an acceptable prognostic biomarker for detecting pathologic cardiovascular conditions such as cardiac hypertrophy, DCM, and myocardial infarction in human patients [20–22]. In humans, titin has been extensively studied as a biomarker of cardiovascular conditions, such as cardiac hypertrophy, DCM, and myocardial infarction. Previous studies demonstrated that alterations in titin levels may signal the onset or progression of these pathologic conditions in HCM, offering prognostic value in clinical settings [29, 31, 32, 37].

However, limited research has explored the significance of titin in the cardiovascular health of animals, particularly cats. The current study on titin levels in cats and their association with echocardiographic parameters represents an important step forward in our understanding of cardiovascular diseases in cats. Specifically, this study demonstrated that, similar to humans, alterations in titin levels in cats correlate with pathological changes such as myocardial hypertrophy and DCM. The findings of this study offer evidence that titin can be linked to structural and functional changes in cats with HCM, as detected through echocardiography.

Previous studies on cardiac microtissues engineered from human-induced pluripotent stem cells have evaluated the pathogenicity of titin gene variants [27, 31]. The findings of these studies indicate that mutant titin protein affects sarcomere insufficiency and impaired adaptive remodeling, resulting in the development of DCM. In addition, Lahmers *et al*. [23] conducted a study on the LV myocardium in animal models, including mice, rats, rabbits, and pigs [23]. This study found that alterations in titin contributed to the impairment of diastolic filling properties and cardiac function during neonatal heart development. Moreover, increased titin concentrations are a candidate molecular mechanism underlying the association between fetal macrosomia and cardiomyocyte/diastolic dysfunction [38]. A previous study by Micaglio *et al*. [38] documented a novel truncating heterozygous variant of the titin gene and described its correlation with DCM in a family with a history of sudden cardiac death. With our findings, these findings indicate that titin may play a role in LV thickening, which initially represents the impact on echocardiographic parameters.

As mentioned above, both biomarkers may be prognostic biomarkers for cardiomyopathy based on their abilities. Nevertheless, further studies in companion animals are required to better understand this clinically relevant gene variant. The roles of Gal-3 and titin in cardiac-related *MYBPC3* gene mutations may provide further insights into the roles of these biomarkers in the pathophysiology of cardiomyopathies. However, this study has some limitations due to the variety of animal breeds and the small number of cats with ventricular hypertrophy.

## Conclusion

Gal-3 and titin had weak correlations with the echocardiographic parameters IVSd and FS. However, titin is more likely to be associated with echocardiographic parameters than Gal-3. In the context of this finding, these biomarkers may have early predictive value for the development of cardiovascular disease in cats.

## Authors’ Contributions

SP: Designed and supervised the study, performed the statistical analysis, produced the tables and figures, and drafted and revised the manuscript. SP, KD, and PS: Analyzed the data. KD: Drafted the manuscript and conducted the ELISA. All authors have read and approved the final manuscript.
